# Increasing adherence to the Mediterranean diet and lifestyle is associated with reduced fecal calprotectin and intra-individual changes in microbial composition of healthy subjects

**DOI:** 10.1080/19490976.2022.2120749

**Published:** 2022-10-13

**Authors:** L. Godny, L. Reshef, T. Sharar Fischler, S. Elial-Fatal, T. Pfeffer-Gik, B. Raykhel, K. Rabinowitz, A. Levi-Barda, TT. Perets, R. Barkan, I. Goren, JE. Ollech, H. Yanai, U. Gophna, I. Dotan

**Affiliations:** a Division of Gastroenterology, Rabin Medical Center, Petah-Tikva, Israel, Affiliated to the Sackler Faculty of Medicine, Tel Aviv University, Tel Aviv, Israel; b The Shmunis School of Biomedicine and Cancer Research, Faculty of Life Sciences, Tel Aviv University, Tel Aviv, Israel; c Biobank, Department of Pathology, Rabin Medical Center, Petah Tikva, Israel; d Gastroenterology Laboratory, Division of Gastroenterology, Rabin Medical Center, Petah Tikva, Israel; e Adelson School of Medicine, Ariel University, Ariel, Israel; f Department of inflammation and Immunity, Lerner Research Institute, Cleveland Clinic, Ohio, USA

**Keywords:** Microbiome, eukaryome, inflammation, nutrition

## Abstract

The Mediterranean diet (MED) is associated with the modification of gut microbial composition. In this pilot study, we investigate the feasibility of a microbiota-targeted MED-based lifestyle intervention in healthy subjects. MED intervention integrating dietary counseling, a supporting mobile application, and daily physical activity measurement using step trackers was prospectively applied for 4 weeks. Blood and fecal samples were collected at baseline, after the 4-week intervention, and at 6 and 12 months. Blood counts, inflammatory markers, microbial and eukaryotic composition were analyzed. Dietary adherence was assessed using daily questionnaires. All 20 healthy participants (females 65%, median age 37), completed the 4-week intervention. Adherence to MED increased from 15.6 ± 4.1 (baseline) to 23.2 ± 3.6 points (4 weeks), p < .01, reflected by increased dietary fiber and decreased saturated fat intake (both p < .05). MED intervention modestly reduced fecal calprotectin, white blood cell, neutrophil, and lymphocyte counts, within the normal ranges (P < .05). Levels of butyrate producers including *Faecalibacterium* and *Lachnospira* were positively correlated with adherence to MED and the number of daily steps. Bacterial composition was associated with plant-based food intake, while fungal composition with animal-based food as well as olive oil and sweets. Increasing adherence to MED correlated with increased absolute abundances of multiple beneficial gut symbionts. Therefore, increasing adherence to MED is associated with reduction of fecal calprotectin and beneficial microbial alterations in healthy subjects. Microbiota targeted lifestyle interventions may be used to modify the intestinal ecosystem with potential implications for microbiome-mediated diseases.

## Introduction

Diet and gut microbiota have emerged as risk modulators of multiple chronic diseases, including inflammatory bowel disease (IBD). The Mediterranean diet (MED) is a plant-based diet, high in fiber and antioxidants, characterized by daily consumption of vegetables, fruits, olive oil, nuts, whole grains, and legumes; and low consumption of ultra-processed and animal-based foods, especially red meat. MED is postulated to be involved in immune-mediated diseases,^1,2^ with recent studies highlighting the potential beneficial role for MED in the management and prevention of IBD.^3–7^

MED has been previously shown to modulate microbiota composition in association with positive health outcomes in healthy and obese subjects, and in individuals with high risk for cardio vascular diseases (CVD).^8–11^ A recent pivotal study demonstrated the effects of two dietary interventions, rich in plant-based fiber or fermented foods, on immune activity,^12^ supporting the emerging field of microbiota-targeted dietary and lifestyle interventions in improving overall health and chronic disease prevention. The accumulating evidence for MED health benefits and effect on the gut microbiota place MED as a promising microbiota-targeted intervention. However, a comprehensive approach that addresses specifically the gut microbiota and other lifestyle factors like physical activity, and uses digital health tools to improve adherence, is lacking.

Here we developed a microbiota-targeted MED-based nutritional education program. We targeted the microbiota by increasing and diversifying plant-based foods and dietary fiber intake, and including fermented foods like yogurt daily. To support lifestyle modifications beyond diet, we provided fitness trackers to monitor for daily steps, physical activity, and lifestyle parameters. Finally, as implementing diet and lifestyle modifications can be challenging with difficulties such as acceptance and the need for personalization, we integrated digital health tools including an interactive mobile application. The aim of this study was to investigate the feasibility of a microbiota-targeted MED-based intervention in increasing adherence to MED lifestyle, while assessing whether increasing adherence to MED lifestyle is associated with changes in inflammatory markers and microbial composition in healthy subjects.

## Results

To assess whether MED intervention is associated with changes in dietary consumption, inflammatory markers and microbial composition, we recruited 20 healthy participants. The median age was 37 years (IQR 31.5–42); the majority were females (13/20, 65%) with a median BMI of 24.7 Kg/m^2^ (IQR 20.7–26.1). Four participants (20%) had a family history of IBD, and four couples shared a household (Table S1). All participants completed the 4-week intervention. Three were excluded from the follow-up due to pregnancy, and one dropped out.

### Diet and lifestyle factors were affected by the MED-based intervention

**Figure 1.**
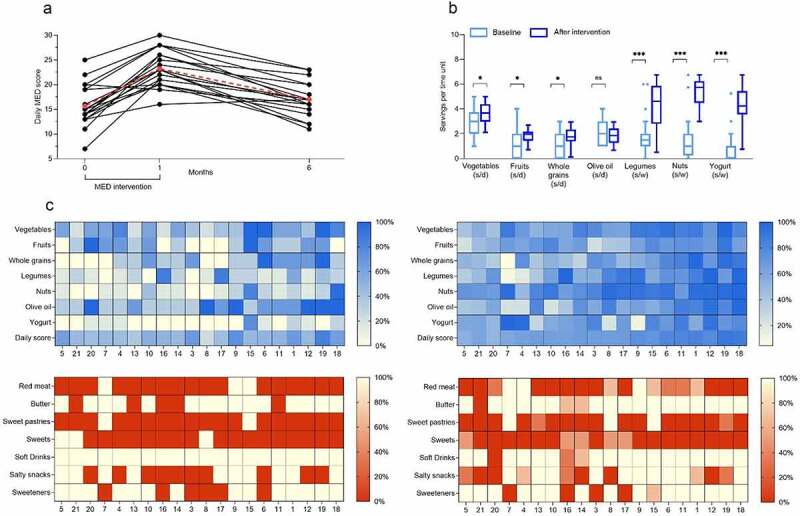
Adherence to Mediterranean diet (MED) based on daily and weekly questionnaires. A: (a) Daily MED score over time based on self-reported daily questionnaire. B: Baseline and average throughout the intervention consumption of each recommended MED component; (s/d: servings per day; s/w: servings per week) C: Heatmaps of individual nutritional data at baseline (left) and during the intervention (right). Each row represents a food component, and each column represents an individual. The intensity of the colors reflects the adherence to MED based on the recommendation in terms of number of servings per day.

We developed the daily MED score (see Methods), a daily, self-reported questionnaire, focused on MED’s recommended foods and lifestyle habits, to assess adherence to the MED intervention. The daily MED score ranges between 0 and 30 and correlated with the previously published MED adherence screeners MEDAS and i-MEDAS (R = 0.77 and R = 0.76, respectively, both p < .001). After 4 weeks of MED-based intervention, the daily MED score increased by 7.6 points, from 15.6 ± 4.1 at baseline to 23.2 ± 3.6 (p < .01, Figure 1a). The consumption of the MED’s recommended food components, including fruits, vegetables, whole grains, legumes, nuts, and yogurt, significantly increased (Figure 1BC) with no significant change in the consumption of MED’s non-recommended foods (Figure 1c). Increased adherence to MED was also reflected by changes in diet composition assessed by 3-day food records. While the total daily caloric intake, as well as protein, carbohydrate, and fat intakes were comparable before and after the intervention, the average dietary fiber intake increased from 24 ± 13 g/d to 30 ± 13 g/d (p = .008). Conversely, we observed a reduction in consumption of saturated fat from 27 ± 9 g/d to 21 ± 6 g/d (p = .03), and trends for reduction in cholesterol and sodium intake (Table S2).

Participants were recommended to increase their daily step number and adopt an active lifestyle. Daily measurements of physical activity, stress, and sleep hours were monitored continuously with the Garmin vívosmart® 4. The personal daily step goal, defined by Garmin, was achieved in 58% of days during the intervention, with an average of 8097 ± 2968 daily steps. The average number of sleep hours was 7.05 ± 0.75, and the average resting heart rate was 61.8 ± 5.6 beats per minute (bpm). Improvement in general well-being was reported by 12 (60%) participants, with 45% reporting softer stool texture and 55%- a change in gas or bloating (Figure S1).

Over time, MED score declined to 17 ± 3.5 and 18.6 ± 5 at 6 and 12 months respectively, and did not statistically differ from baseline; however, after 12 months follow-up, 16/16 (100%) of study participants still reported an increase in consumption of at least one of MED’s recommended foods, mainly olive oil, vegetables, fruits, nuts, and yogurt (Figure S2), and 10/16 (62%) reported that participation in the study affected the dietary choices of their household members.

### Increasing adherence to MED is associated with reduction in inflammatory markers

**Figure 2.**
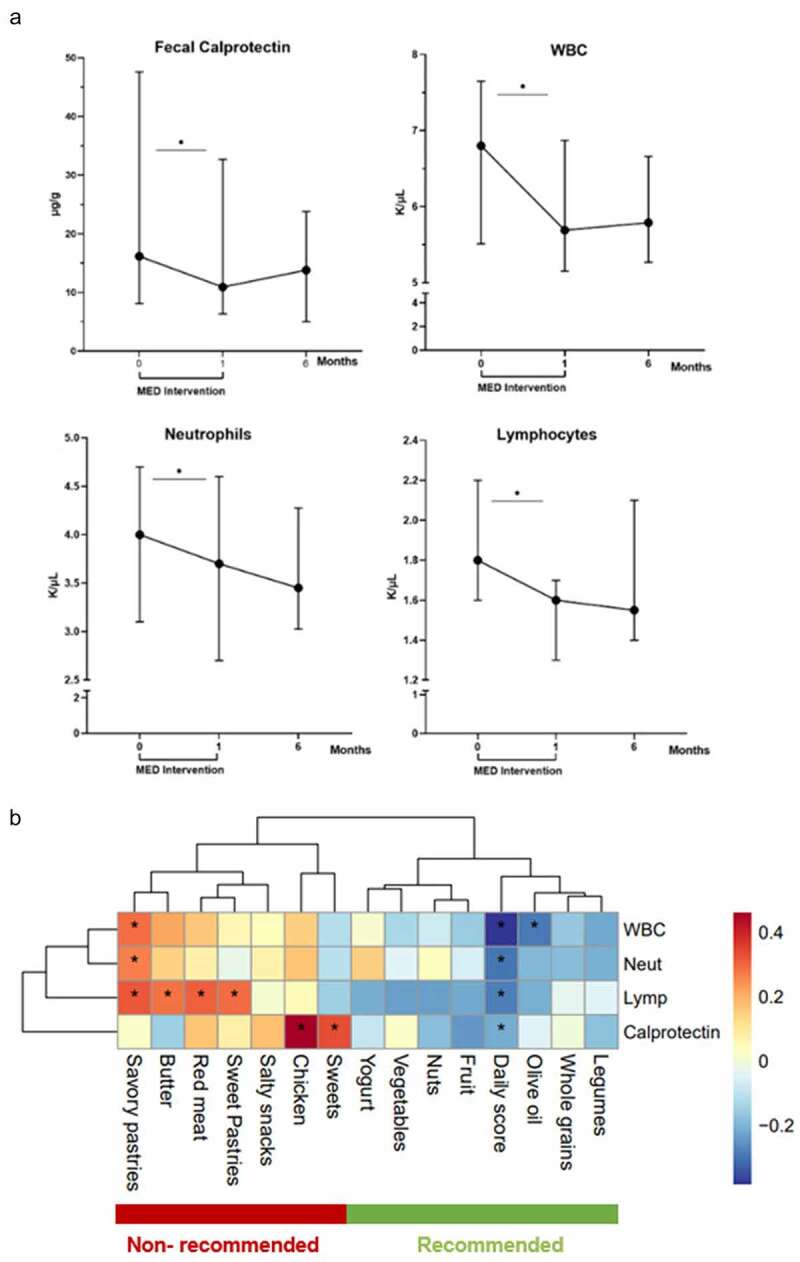
Increasing adherence to MED is associated with reduction in inflammatory markers. A: Cell blood counts and fecal calprotectin before and after the 4-week intervention and after a 6 month follow up. B: Correlation map showing the interactions between different food components and inflammatory markers that decreased after the intervention.

Comprehensive laboratory analyses were performed pre and post intervention. Body weight, CRP, serum vitamin levels and lipid profiles were comparable before and after the intervention (Table S3). At 4 weeks, the fecal inflammatory marker calprotectin significantly decreased from 16.5 μg/g (IQR 8–47) to 10.9 μg/g (IQR 6–33, p < .05). This decrease was also associated with reductions in white blood cells from 6.8 K/μL (IQR 5.5–7.6) to 5.7 K/μL (IQR 5.1–6.8), neutrophils count from 4 K/μL (IQR 3.1–4.7) to 3.7 K/μL (IQR 2.7–4.6) and lymphocytes count from 1.8 K/μL (IQR 1.6–2.2) to 1.6 K/μL (IQR 1.3–1.7), all within the normal ranges (p < .05, Figure 2a). Interestingly, fecal calprotectin, WBC, neutrophils and lymphocytes levels were inversely correlated with adherence to MED and with the average intake of MED’s recommended foods and positively correlated with MED’s non-recommended foods (Figure 2b). Of note, at 6- and 12-month follow-up, levels of fecal calprotectin, WBC, neutrophils, and lymphocytes were comparable to baseline.

### Overview of bacterial (16S) and eukaryotic (18S) microbiome

We next explored the contribution of clinical, demographic, dietary, and lifestyle factors to microbial variation. Microbial and eukaryal profiles were obtained from fecal samples by deep amplicon sequencing of the 16S/18S rRNA gene, respectively. DADA2 pipeline yielded a median of 11,283 clean sequences per sample for 16S, and 6170 for 18S. Subject identity accounted for 72% of microbial variation (PERMANOVA, p = .001), and after controlling for personal identity, the intake of legumes, whole grains and nuts, accounted for 1.6–3.3% of microbial variation (q = 0.064–0.16, Table S4). These PERMANOVA R values are within the range previously reported for dietary components.^11^ Importantly, a similar R value was obtained for the daily MED score alone (q = 0.064, R = 3.0%). As expected, the eukaryome was far less diverse than the bacterial microbiome, as well as unstable over time and lacking a strong personal signature (Figure S3). Different dietary association patterns were revealed for the eukaryome; with a highly significant effect (R = 0.11, p = .009, q = 0.13; Adonis. Table S4) observed for sweet consumption.

Of note, two of the 4 couples sharing a household portrayed microbiomes that were more similar to each other than to other study participants and this was correlated with higher adherence to MED and with similarities in their diet (Figure S4).

### Differential effects of diet and lifestyle on the microbiome and eukaryome

**Figure 3.**
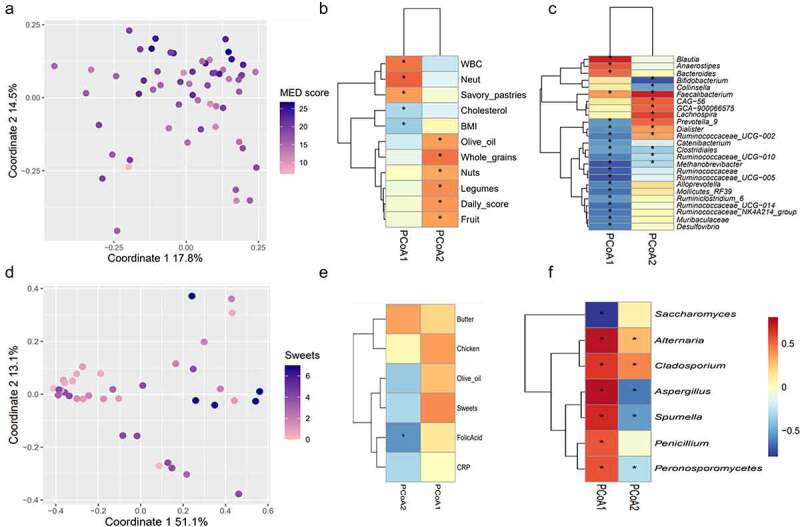
Factors affecting the bacterial and eukaryotic microbiomes. A: PCoA (Bray-Curtis); each point represents a single sample and is colored by the corresponding daily MED score. B-C: Spearman’s correlations between the coordinates of each of the first two PCoA axes and clinical/dietary variables (b) or relative abundance of bacterial genera (c). D-F: Same as A-C, for eukaryotic microbiome; points in (d) are colored by sweets consumption. Criterions for feature inclusion in heatmaps were:(B) q < 0.2 and R>[0.3|; (e) p < .05 and R>|0.3|; (c,f) q < 0.2 and R>[0.5|. For all panels, correlations with q values <0.2 are marked by asterisks.

Host–microbiome interactions were explored by applying Principal Coordinate Analysis (PCoA) (Figure 3a, d; Methods). The coordinates of each sample along each of the first two PCoA axes were extracted, and correlated against dietary and clinical parameters (Table S5). Notably, coordinates of the 1^st^ PCoA axis correlated with blood neutrophils and WBC (p = 6*10^−5,^ 4*10^−5^; q = 0.006, 0.01; R = 0.51,0.47, respectively), and, inversely with BMI and cholesterol (p = .005,0.02; q = 0.04,0.13; R = −0.35, −0.3, respectively). Conversely, coordinates of the 2^nd^ axis correlated with MED’s recommended foods, and with the daily MED score (p:0.0015 to 0.007; q:0.004 to 0.04; R:0.34 to 0.47; Figure 3b). Predictably, since the PCoA was based on a weighted bacterial composition measure (Bray-Curtis), both axes correlated with multiple bacterial genera (Figure 3c).

In contrast, for the eukaryome, the 1^st^ principal PCoA axis correlated mainly with sweets (p = .01, R = 0.41) and with chicken (p = .02, R = 0.36). The 2^nd^ axis correlated with serum folic acid (p = .0005, R = −0.5, q = 0.016); and to a weaker degree with olive oil and butter (p = .02, R = −0.37 and p = .03, R = 0.35, 0.3 < q < 0.36, Figure 3d-f, Table S6).

**Figure 4.**
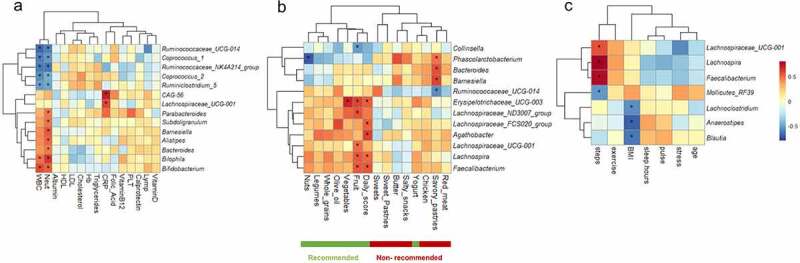
Post-intervention (single time point) microbial correlations with clinical (a), dietary (b) or lifestyle/demographic (c) variables; q values <0.2 are marked with asterisks. Features for which all correlation q values exceeded 0.2, and/or all correlation R values were <|0.3| are not shown.

To identify specific interactions between microbial genera and dietary, clinical, or lifestyle variables, pairwise Spearman’s correlation was applied using data from the post-intervention time point (Table S6). Highly correlated microbial-metadata pairs are shown as heatmaps, clustered according to co-interaction patterns (Figure 4a-c). WBC and neutrophils were most strongly correlated to multiple bacterial genera; with strong negative correlations (R = 0.6, p < .004, q < 0.1) to *Ruminococcaceae_NK4A214_group* and *Ruminococcaceae_UCG-01*4 (Figure 4a). Bacterial–dietary interactions formed two distinct co-occurrence clusters (Figure 4b). Interestingly, the daily MED score and MED’s recommended food components formed a single cluster, driven by strong positive correlations to multiple beneficial genera of the *Ruminococcaceae* and *Lachnospiraceaea* families. *Lachnospira* and *Faecalibacterium* also correlated with the average number of daily steps (R = 0.8, q = 0.0001; R = 0.76, q = 0.0002; respectively, 4C). These taxa tended to be inversely correlated with age, pulse, and stress. We next fitted multiple linear regression models, in which steps, age, gender, BMI, and fruit intake were all set as explanatory variables. The association with steps remained highly significant for both models (p = .0008 for *Lachnospira*; p = .006 for *Faecalibacterium*; Figure S5), implying an interaction that is independent of diet.

Conversely, MED ‘s recommended plant-based components, except for olive oil, had little or no effect on eukaryotic taxa, while red meat, chicken and sweets exerted the strongest effects (Figure S6). These results suggest that MED’s dietary components and lifestyle variables may modify bacterial composition toward a beneficial *milieu*, while MED’s non-recommended food components contribute to eukaryal modification.

### Increasing adherence to MED is associated with increased loads of beneficial bacteria

Finally, to control for individual variability in baseline dietary intake and adherence to the intervention, we constructed differential ‘delta microbiome’ (post-intervention – pre-intervention relative abundances, for each genus across all subjects) and ‘delta nutritional’ (post-intervention – pre-intervention daily intakes, for each dietary component across all subjects) matrices (Methods, Table S6).

Changes in the dietary intake of sweets were most strongly correlated to microbial changes, with *Blautia* increasing and *Bacteroides* decreasing in response to increased sweets consumption (R = 0.69, p = .0007, q = 0.01, R = −0.62, p = .003, q = 0.026, Fig. S6). Changes in yogurt and salty snacks consumption also affected multiple bacterial genera, specifically increasing abundance of *Bacteroides* with increasing yogurt consumption, and increasing abundance of *Lachnospira* and *Faecalibacterium* with decreasing consumption of salty snacks, albeit to a lesser degree (R = 0.46, p = .042, q = 0.21, r = −0.44, p = .05, q = 0.24, R = −0.4, p = .07, q = 0.24, Table S6).

**Figure 5.**
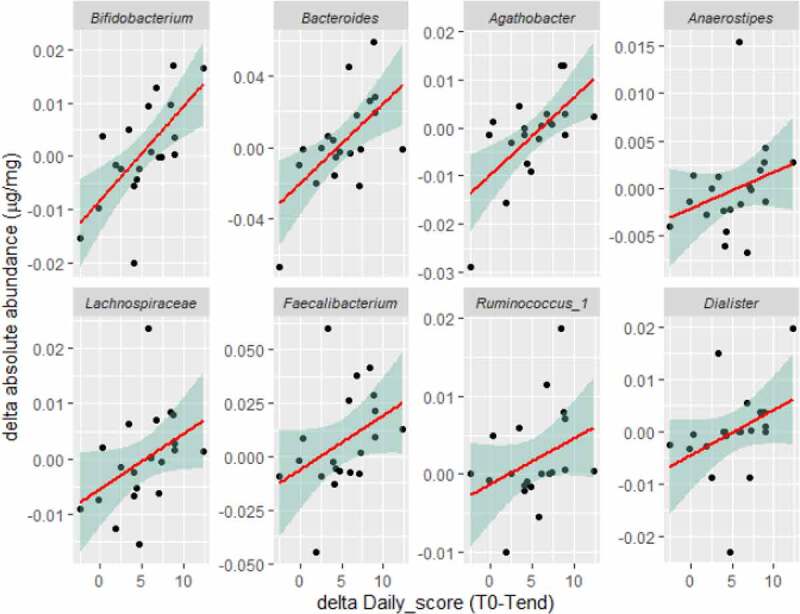
Personal changes in the daily MED score post-pre intervention correlate with the changes in bacterial absolute abundances. Spearman’s correlation; correlations with p < .05, q < 0.2 and R > 0.4 are shown.

Standard microbiome workflows focus on compositional (relative abundances) only. Yet, reduced bacterial absolute abundances, observed in patients with IBD,^13^ highlight the importance of bacterial densities. We thus estimated the bacterial load per sample (µg bacterial DNA/mg stool) using qPCR,^13^ and transformed bacterial relative abundances to absolute abundances (see Methods). A ‘delta microbial load’ matrix was then constructed (post-intervention – pre-intervention *absolute* abundances, for each genus across all subjects) and correlated to the personal change in daily score index (daily score post-intervention – daily score pre-intervention). Multiple bacterial genera, all considered beneficial gut commensals, portrayed absolute post-intervention increases significantly (p < .05, q < 0.2; all with R > 0.4) correlated to increases in MED daily score (Figure 5), indicating that increased adherence to MED promotes increased microbial loads.

## Discussion

This pilot study shows that a 4-week microbiota-targeted, MED-based intervention is feasible and increases adherence to MED lifestyle while reducing levels of the intestinal inflammatory marker fecal calprotectin in healthy subjects. Microbial variation was attributed to host, dietary, and lifestyle factors and increasing adherence to MED diet and lifestyle was associated with increased absolute abundances of beneficial microbial taxa.

In the era of popular exclusion diets, MED differs by emphasizing foods to include in the diet, specifically fruits, vegetables, whole grains, legumes, nuts, and olive oil on a daily basis. The daily MED score, developed in this study, assesses only MED’s recommended foods and lifestyle behaviors, formatted as a daily checklist. It provides immediate feedback, thereby helping in creating and preserving lifestyle behavioral changes. The increase in daily MED score achieved throughout the intervention was associated with improved diet composition, increased dietary fiber intake, and reduced saturated fat and sodium intake, reflecting a reduction in animal-based and ultra-processed foods. In order to target microbiota alterations, we focused on increasing and diversifying dietary fiber intake by scoring the daily consumption of fiber-containing foods, color diversity in the diet and the intake of yogurt as a source of fermented foods, in compliance with recent reports that fermented foods had a greater effect on microbiome than a high fiber diet.^12^ Interestingly, softer stool texture and changes in gas or bloating reported by the participants might be associated with increasing fiber and yogurt intake. Importantly, adherence to MED increased significantly throughout the intervention but decreased over time, highlighting the need for a long-term maintenance strategy.

Within the 4-week MED intervention, we detected a reduction in the intestinal inflammatory marker fecal calprotectin, expanding our previous report that adherence to MED is inversely associated with fecal calprotectin in patients with IBD.^6^ Additionally, we observed significant reductions in WBC, leucocyte, and neutrophil counts, all within the normal ranges that were inversely correlated with overall adherence to MED and with MED’s recommended foods. This may suggest that higher WBC, lymphocytes, and neutrophil levels, within the normal ranges, may be biomarkers for an unhealthy lifestyle. This hypothesis is in line with previous findings showing adherence to MED and its recommended foods is inversely associated with WBC,^14^ lymphocytes,^15^ and risk of developing leukocytosis or leukopenia in high CVD-risk individuals.^16^ WBC is a broadly used marker of acute systemic inflammation that has also been investigated as a cellular biomarker of chronic low-grade inflammation, with the potential to serve as a risk marker for cancer, CVD and mortality.^17–19^ Notably, WBC and neutrophil levels were correlated to multiple bacterial genera, including fiber-degrading gut bacteria. This might demonstrate the interplay between host dietary patterns, microbial composition and low-grade inflammation in healthy subjects.

Dietary-bacterial interactions in this study formed two distinct co-occurring clusters: a major cluster linking all plant-based foods exclusively with diverse genera of the Clostridiales order, and a minor one linking animal-based and ultra-processed foods with *Bacteroides, Barnesiella, Phascolarctobacterium*, and *Collinsella*, some of which were previously associated with high-fat diet.^20,21^ Interestingly, *Collinsella*, which was reduced in 16/20 participants in this study (paired-Wilcoxon p-value: 0.03), was shown to play a mediatory role in WBC decrease following MED intervention in the PREDICT cohort,^15^ as well as being positively correlated with processed foods and negatively correlated with vegetable consumption.

Unlike the diverse array of bacteria colonizing the gut, few eukaryotic microorganisms are considered possible gut colonizers.^22^ Here, eukaryotic composition was dominated by *Saccharomyces, Penicillium*, and *Aspergillus*, and associated mainly with sweets and animal-based foods. We previously demonstrated that starch intake is associated with the abundance of *Candida albicans* in patients with IBD.^23^ In this cohort, *Candida* prevalence was low (15%; mean RA 0.003), yet, intriguingly, sweets remained a significant factor affecting eukaryal composition. Coupled to our observation that sweets consumption affected the bacterial genera *Blautia* and *Bacteroides*, these findings suggest sugars, despite being rapidly absorbed by the host, may exert a substantial effect on the gut microbiome.

Interestingly, the microbial genera *Lachnospira* and *Faecalibacterium* were associated with adherence to MED and strongly correlated with the average number of daily steps. While previous reports linked between physical activity and increased richness and abundance of beneficial bacteria,^24,25^ this report highlights the direct association with the number of daily steps. Importantly, we had previously reported that fruit consumption is associated with *Lachnospira* and *Faecalibacterium*^26^ relative abundances, and dietary fiber intake associated with levels of butyrate producing genes,^27^ in patients with IBD after pouch surgery, implying potential similarities in host-diet-microbiome patterns observed in healthy subjects and patients with IBD.

Increasing adherence to MED lifestyle was associated with increased abundances of fiber degrading and butyrate producing bacteria, as was previously described.^8,9,28,29^ Host-beneficial microbial activities, such as fiber degradation, butyrate-production and colonization resistance are likely to be biomass-dependent; and reduced bacterial biomass has been reported in IBD.^13,30^ Here we demonstrate that increased adherence to MED correlates with increased biomass of many beneficial gut microbial genera, such as *Faecalibacterium, Lachnospira*, and *Bifidobacterium*, suggesting further MED benefits for patients with IBD. Additionally, our results are consistent with a recent study in first-degree relatives of patients with CD, demonstrating an association between MED-style dietary cluster, increased abundance of fiber degrading bacteria including *Faecalibacterium* and lower levels of fecal calprotectin.^31^

The study has several strengths: first, the comprehensive approach to improve lifestyle behaviors using a nutritional education program with dietary counseling and a digital application providing real-time feedback and support. Second, we provided step trackers that enabled us to monitor physical activity, number of daily steps, stress levels, and sleep hours, while setting personal daily goals to gradually increase activity levels. Using digital health interventions may advance healthcare delivery and assist not only in improving lifestyle in healthy participants but also in managing chronic diseases that require frequent medical care and personalized guidance.^32–34^ Finally, microbiome analysis included an assessment of both relative and absolute abundances, and alongside bacterial microbiome we also assessed eukaryal microbiome to deeper understand host-diet-microbiome interactions.

We acknowledge several limitations: first, considering the small sample size and lack of a control group, our findings are limited to associations. This comprehensive lifestyle intervention makes it difficult to determine whether the microbial changes were driven by dietary changes, physical activity, or the psychological benefits of participating in a study. In the absence of a control group, there is no guarantee that the effects would have occurred without the intervention. Yet, our results are in line with observational findings associating adherence to MED with increased abundances of beneficial microbial groups and with lower levels of inflammatory markers.^11^ Second, this was a short-term intervention and we show that adherence to MED was not maintained over time. However, even with this short-term intervention we were able to demonstrate lasting effects in dietary intake. Longer term dietary and lifestyle maintenance strategies are required to preserve their consequent effects on health outcomes.

In conclusion, a microbiota-targeted MED-based intervention may contribute to increased adherence to MED, improved diet quality and modification of the gut ecosystem by increasing abundances of beneficial and butyrate producing bacteria, and reducing intestinal markers of inflammation. This approach might be used not only to benefit healthy subjects, but also in the management and prevention of microbiota-associated diseases including IBD. Intervention studies assessing this approach in patients with IBD are ongoing.

## Methods

### Study participants and design

This feasibility pilot study was conducted as part of a project evaluating the effect of dietary intervention in patients with IBD (NCT04082559, biomarker-based multi-disciplinary team approach, Bio-MDT). Healthy adults were recruited at the Rabin Medical Center, Israel. All provided written informed consent (0129–19- RMC), completed a screening visit during January 2020, and were recruited to a 4-week intervention between 09.01.2020 and 08.02.2020 and a one-year follow-up. Participants were excluded if they had CVD, diabetes, IBD or any malignancy. Female participants were excluded from the study or follow-up if they were pregnant.

### Intervention

We developed a microbiota-targeted, MED-based intervention, aiming to increase adherence to MED. We focused on increasing the intake of MED’s recommended foods, including fruits, vegetables, whole grains, legumes, nuts, and olive oil, and decreasing the intake of MED’s non-recommended foods, including animal-based, especially red and processed meat and ultra-processed foods (processed pre-packed foods that contain food additives and high amounts of sugar, salt or saturated fat-like sweets, pastries, snacks and soft drinks). In addition, we focused on increasing and diversifying sources for dietary fibers and included fermented foods like yogurt daily. The interactive program comprised dietary counseling, supported by a mobile application developed by us for this program to assess adherence, provide MED-based information and recipes, and an interactive blog in which participants could comment and upload photos, sharing their experience with other participants. Study dietitians were available on the online chat of the MED study application and provided continuous support and feedback of the participant progress throughout the intervention. In addition, fitness trackers were provided to monitor for daily steps, physical activity, and lifestyle parameters.

### Dietary adherence assessment

Adherence to MED was assessed daily using a self-reported MED score, comprised of 11 questions developed by us to assess MED’s recommended diet and lifestyle behaviors. The daily MED score ranges between 0–30 points and scores the daily intake of fruits, vegetables, olive oil, whole grains, legumes, nuts, yogurt, and water. In addition, we assessed the food color diversity by scoring each of the food colors (red, green, orange, white, yellow, and purple) consumed on the same day. We also scored physical activity and achievement of the personal daily step goal. We correlated the daily MED score with previous validated MED adherence screeners, including the Mediterranean Diet Adherence Screener (MEDAS)^35^ and the Israeli Mediterranean Diet Adherence Screener (i-MEDAS).^36^ The daily MED score was calculated (Table 1) and presented immediately to participants so they could track their progress throughout the intervention. In addition, participants were assessed by the study dietitians on a weekly basis regarding both MED’s recommended and non-recommended foods and received personal MED-based recommendations and recipes based on their preferences. We evaluated dietary intake with food frequency questionnaires and 3-day food records collected at baseline, after the 4-week intervention and 6- and 12-months during follow-up.

**Table 1.** Daily MED score components.

Component	Serving size	Recommended number of servings per day	Scoring	Scoring range
Vegetables	1 medium size	≥5	1 point for each serving	0–5
Fruits	1 medium size	≥3	1 point for each serving	0–3
Food colors	-		1 point for each color (red, green, yellow, orange, white, purple)	0–6
Olive oil	1 tablespoon	≥3	1 point for each tbsp	0–3
Nuts and seeds	Handful/ 1 tablespoon of tahini or natural nut spread/butter	≥1	1 point = 1 handful	0–1
Legumes and soy products	½ cup of cooked legumes/2 tablespoons of home-made hummus/tofu	≥1	1 point = 1 serving	0–1
Yogurt	1 cup	1	1 point = 1 serving	0–1
Whole grains	½ cup of cooked whole grains/1 slice of whole grains bread	≥3	1 point for each serving	0–3
Water	1 glass	≥5	1 point for each glass	0–5
Daily step goal		Personal goal	1 point = yes	0–1
Exercise			1 point = yes	0–1
**Daily MED score**				**0–30**

### Fitness trackers

At baseline, participants received a fitness tracker, the Garmin vívosmart® 4 (Garmin Ltd, Schaffhausen, Switzerland), to provide objective continuous measures of daily steps, heart rate, sleep hours and stress level calculated by heart rate variability, which refers to the time elapsed between heartbeats, as previously described.^37,38^ Furthermore, the activity tracker was used to motivate participants to increase daily steps, active time and to achieve daily step’s goal that shifts up or down based on activity level of previous days.

### Clinical and laboratory outcomes

General wellbeing, gastrointestinal symptoms such as gas and bloating and changes in stool frequency and consistency were evaluated using a self-reported questionnaire and Bristol stool chart. Before and after the 4-week intervention and at 6 and 12 months, body mass index (BMI) was measured, and blood and fecal samples were collected. Serum samples were assayed for total cholesterol, low-density lipoprotein (LDL) cholesterol, high-density lipoprotein (HDL) cholesterol, triglycerides (TG), vitamin B12, vitamin D, folic acid, C-reactive protein (CRP) and complete blood count (CBC) including WBC, lymphocytes, neutrophils and platelets. Fecal samples were collected and stored at −20°C until transfer to central laboratory. Fecal calprotectin levels were assessed using the LIAISON® Calprotectin assay and performed on the LIAISON® XL chemiluminescence analyzer according to the manufacturer’s instructions (Diasorin, Saluggia, Italy).

### Fecal sample collection, DNA extraction, and sequencing

Stool samples were collected at RMC, aliquoted and stored at −80°C until testing. Microbial analysis (16S) was performed on 59 stool samples collected at three time points: the first (T0), and last day of the intervention (Tend), and at 6 months post-intervention. Analysis of eukaryotic microbiome (18S) was performed on T0 and Tend time points. DNA extraction and deep amplicon sequencing of the bacterial 16S rRNA gene (V4 region) and eukaryal 18S rRNA gene (first 400 bp) were performed at HyLabs (Rehovot, Israel), Next-Generation Sequencing (NGS) unit and described in detail in Supplementary Methods.

### Microbiome data analysis

Sequencing data were processed using custom R scripts and the DADA2^39^ R package as described in Supplementary Methods. Permutational multivariate analysis of variance (PERMANOVA) analyses were conducted using adonis from vegan^40^ R package; for 16S bacterial analyses, subject identity was set as a blocking factor using the ‘strata’ option. In this and all correlation analyses, multiple hypotheses were controlled using the FDR method; FDR-adjusted p-values are reported throughout the text as ‘q-values’. Linear mixed-effects models for *Lachnospira* and *Faecalibacterium* were constructed using R package lmerTest,^41^ setting subject identity as random effect. To approach normal data distributions, microbial features were first log-transformed (following imputation of zero values with half the minimal value observed for the feature). Full model description and outputs are shown in Supplementary Materials. Correlation analysis was conducted using Spearman’s method. To reduce spurious correlations, rare features (prevalence<10) were filtered before correlating. Delta relative and absolute abundance matrices were constructed by subtracting, per-subject, the abundance of each bacterial genus at T0 (pre-intervention) from Tend (post-intervention). To reduce spurious correlations, only genera whose delta abundance differed from 0 in at least 10 subjects, and in which the change exceeded 1% in at least 1 subject, were retained for correlation analysis. R packages ggplot2^42^ and pheatmap^43^ were used for plotting.

### Estimation of bacterial absolute abundances

Absolute amounts of bacterial DNA per sample were estimated using QPCR as presented in^13^ and described in detail in Supplementary Methods. A custom R script was used to multiply, per sample, the relative abundance of each genus by the absolute bacterial load, thereby translating relative bacterial abundances to absolute ones.

The authors confirm that the data supporting the findings of this study are available within the article or its supplementary materials.

## Supplementary Material

Supplemental Material
