# Supplementary material for: Increasing adherence to the Mediterranean diet and lifestyle is associated with reduced fecal calprotectin and intra-individual changes in microbial composition of healthy subjects
Source: Gut Microbes. 2022 Oct 13;14(1):2120749. doi: 10.1080/19490976.2022.2120749 (PMC9578447; doi:10.1080/19490976.2022.2120749)
Supplement: Supplemental Material [file KGMI_A_2120749_SM5833.zip › Pilot_MED_supp_13082022.docx]

**Increasing adherence to the Mediterranean diet and lifestyle is associated with reduced fecal calprotectin and intra-individual changes in microbial composition of healthy subjects
Supplementary Materials:**

**Supplementary Figures:**


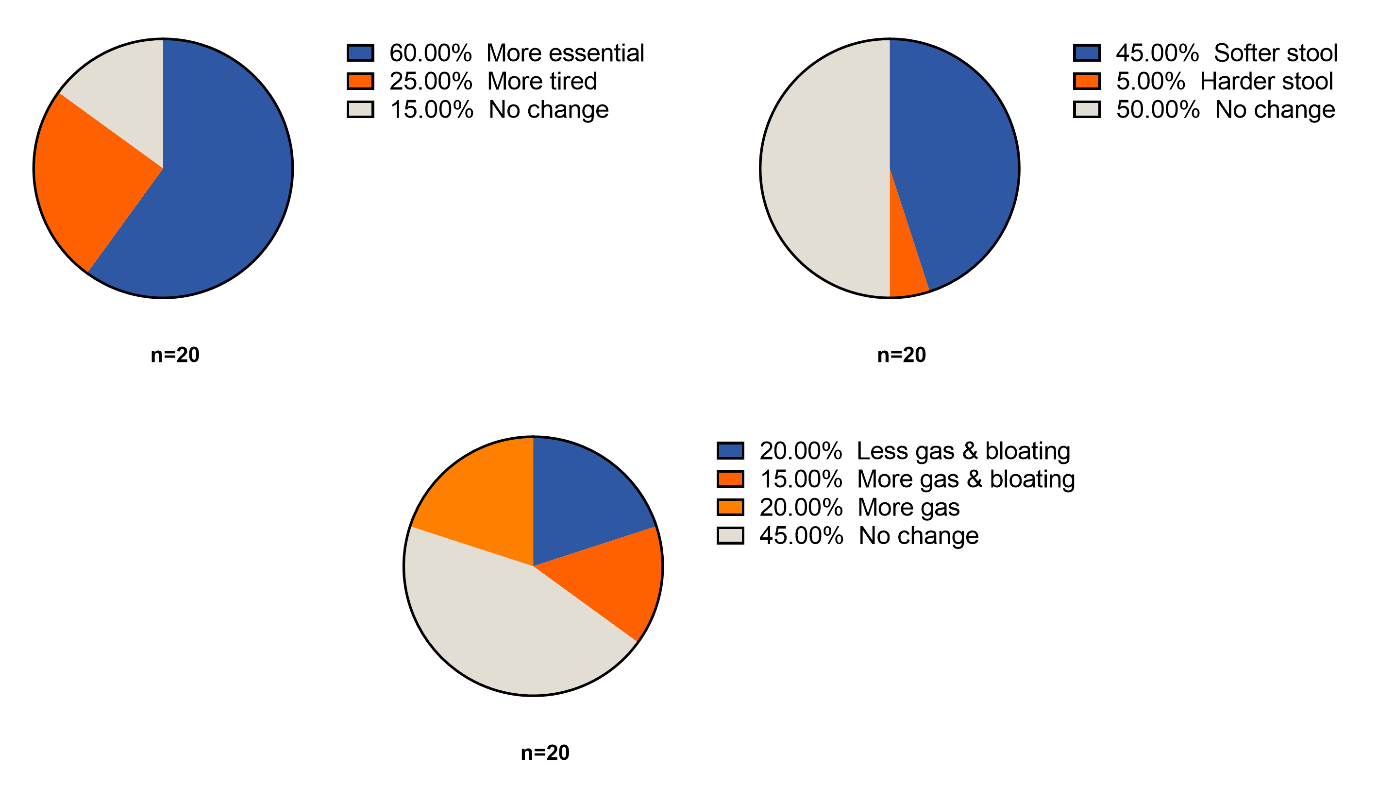


**Figure S1**: Changes in wellbeing, stool consistency and GI symptoms after the 4-week intervention.


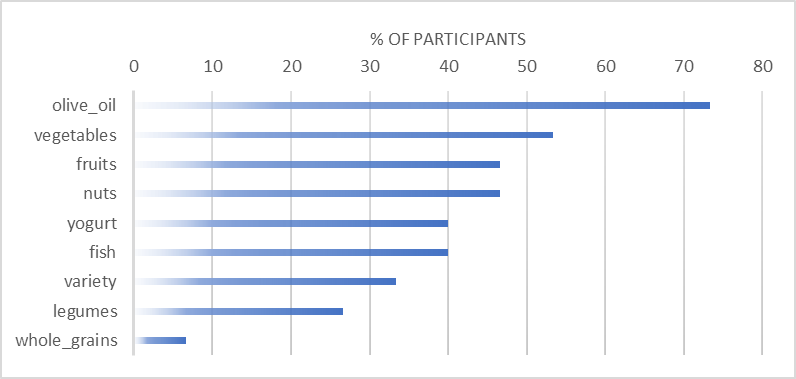


**Figure S2** long term dietary changes at 12 months follow up (n=16)


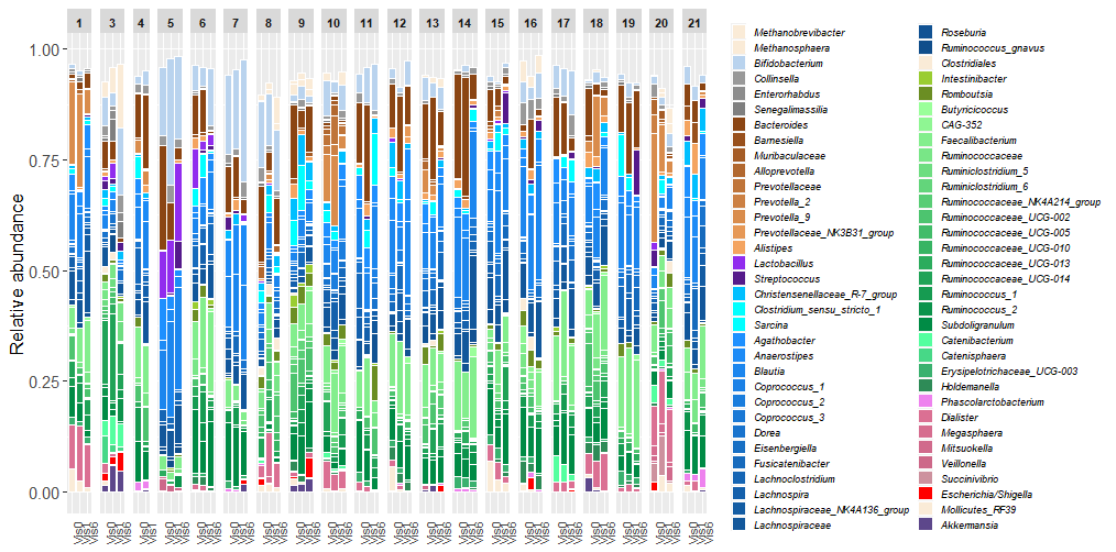
A.

B.


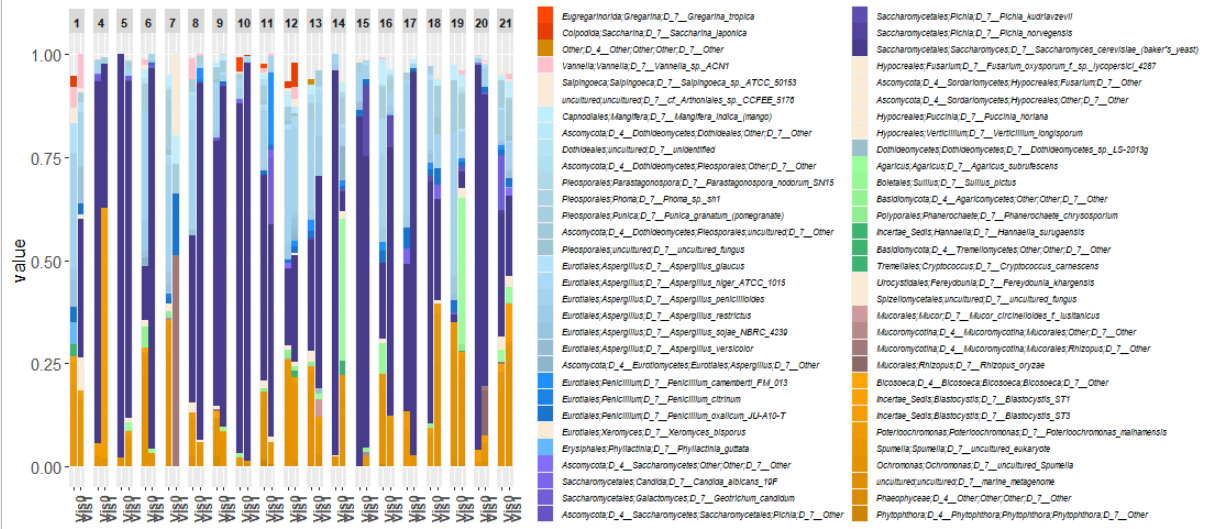


**Figure S3.** Microbiome composition per sample. A. 16S bacterial composition; B. 18S eukaryal composition. Relative abundances per sample are shown, and grouped according to subject ID. Genera not passing a threshold of 4% /1% (16S/18S, respectively) in at least one sample are not shown. Color coding represents phylogenetic assignment. For 16S: Actinobacteria in varying shades of gray, Bacteroidetes in brown, Firmicutes in cyan (for *Clostridiales*), blue (for *Lachnospiraceae*), green (for *Ruminococcaceae*, *Erysipelotrichaceae*, and *Peptostreptococcacea*), and pink for *Veillonellaceae;* Proteobacteria in red and Akkermensia dark purple. For 18S: Basidiomycota in shades of green, Ascomycota in blue with *Saccharomycetales* in purple; and non-fungal unicellular eukaryotes in red and orange.


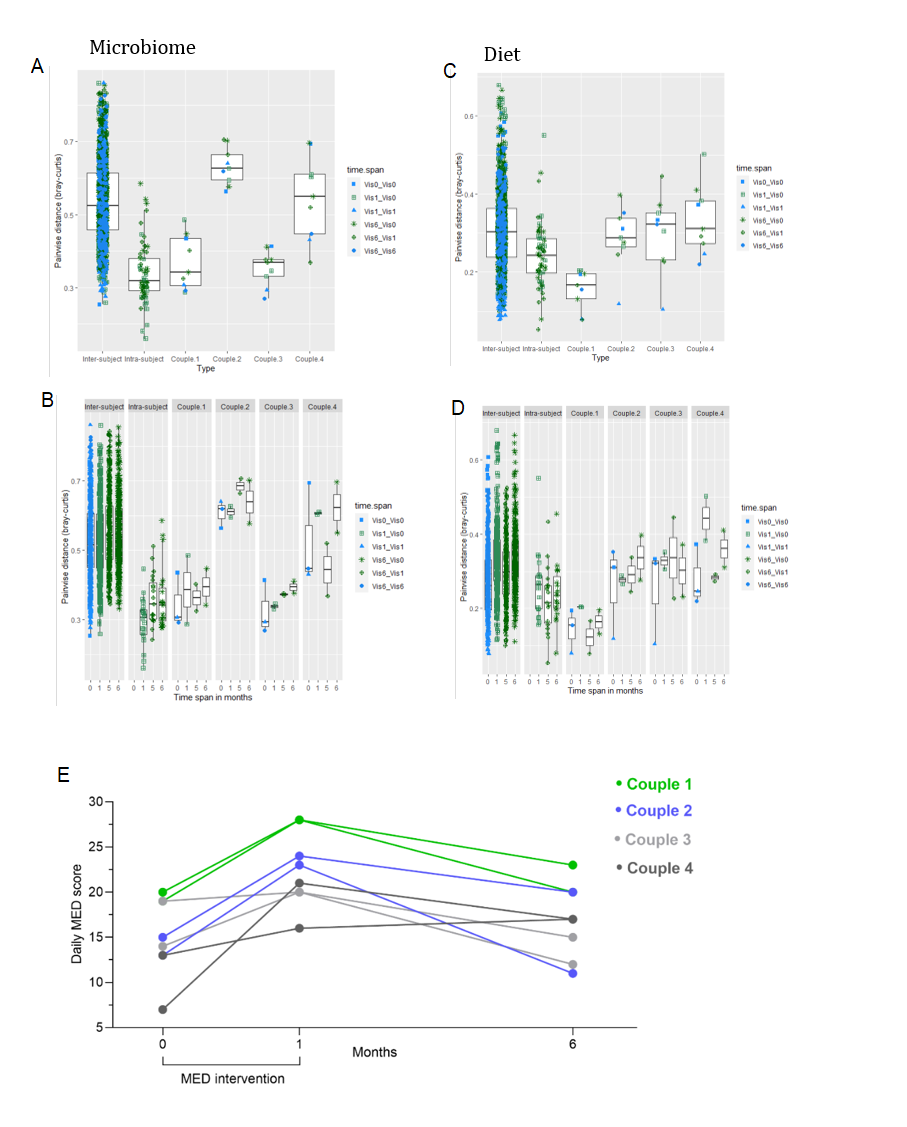


**Figure S4**: Couples sharing a household portrayed microbiomes more similar to each other than to other study participants and this was correlated with higher adherence to MED and with similarities in their diet. A,C: Pairwise Bray-Curtis distances of the microbial genus-level relative abundance table. Each point represents microbial distance between 2 samples. Points are colored according to the visits at which samples were procured, and arranged on X-axis according to origin: Inter-subject (each sample of the pair obtained from a different subject); intra-subject (both samples of the pair obtained from the same subject); Couple 1, 2, etc are specific cases of inter-subject distances, where paired samples were obtained from the same couple. B,D; Similar representation as in A,C, based on dietary intake rather than microbial composition. E. Changes in MED score per each couple across the intervention.


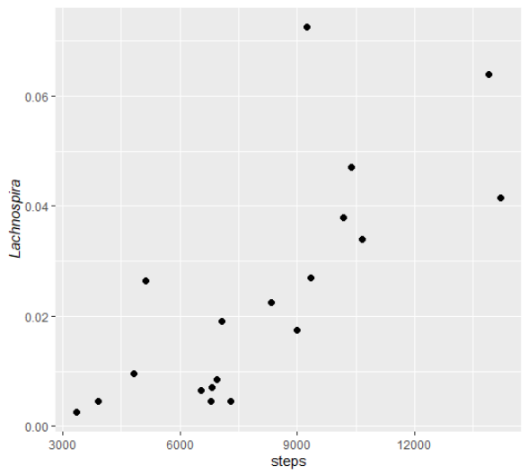

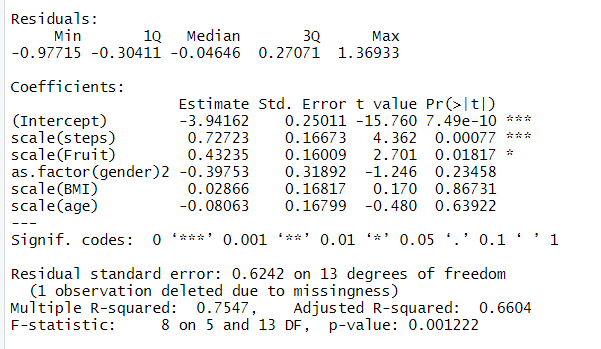


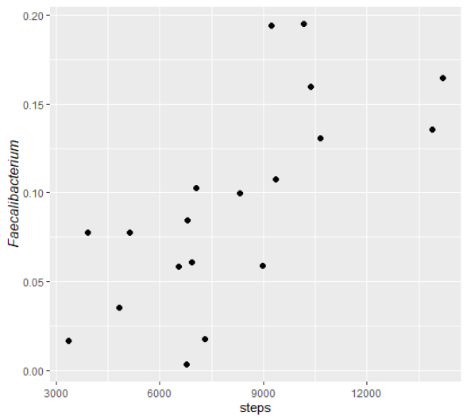


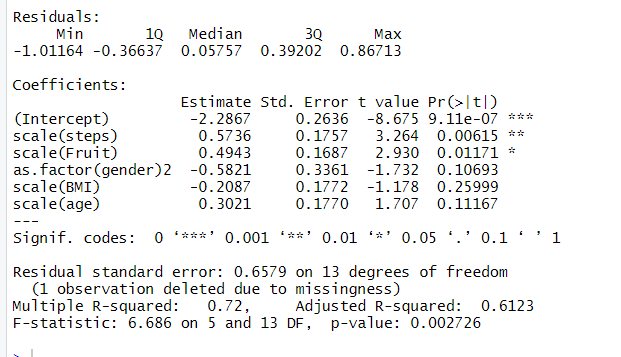


**Figure S5**: Regression analysis for the genera *Lachnospira* (top) and *Faecalibacterium* (bottom). Full output of lm function (base R) is shown. Scatterplots of the relative abundance of these genera at the end of the intervention vs. average daily steps recorded during the intervention are shown. All numeric covariates were scaled to count for different ranges of measuring units; bacterial relative abundances were log-transformed as explained in Methods


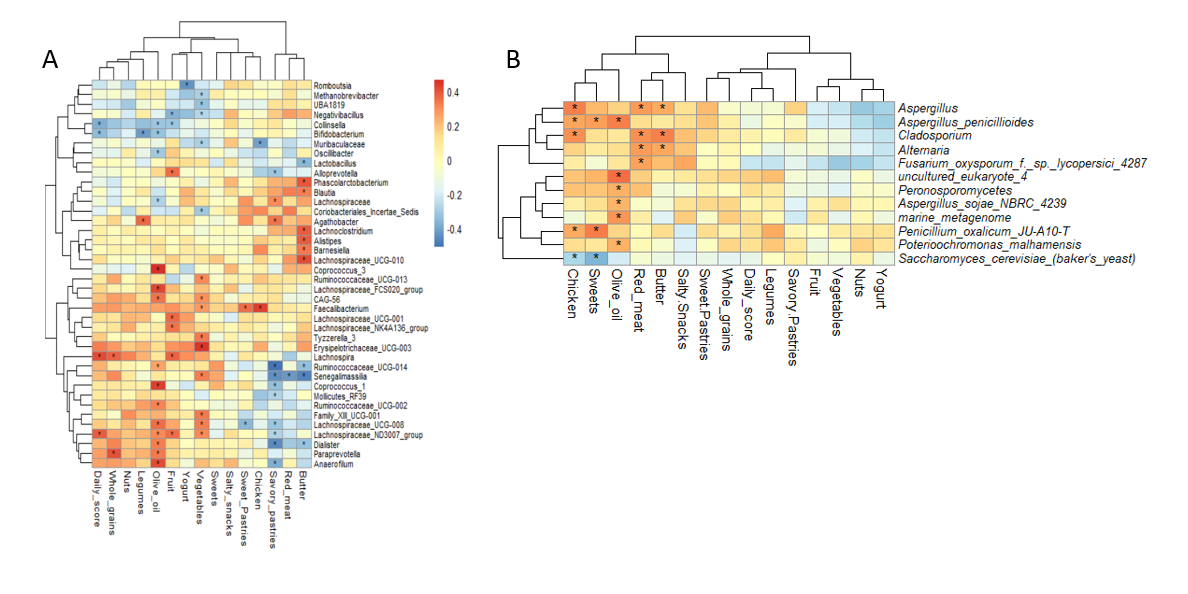


**Figure S6**: Associations between dietary factors and bacterial (A) and fungal (B) genera across the entire dataset (3 time points). Spearman’s correlation analysis was performed using genera relative abundances against dietary intake data. Genera appearing in fewer than 10 samples were excluded to minimize spurious correlations. Spearman’s correlation coefficient (R) is shown; interactions with q<0.2 marked with asterisk. Only genera that had at least 1 interaction with q<0.2 and at least 1 interaction with |R|>0.35 are shown.

**Supplementary Tables:**

**Table S1: Characteristics of the subjects of the study**

| **Characteristics** |  |
| --- | --- |
| **Gender**: females, n [%] | 13 [65%] |
| **Age** (years), median [IQR] | 37 [31.5-42] |
| **Origin, n [%]** |  |
| Ashkenazi | 12 [60%] |
| Non-Ashkenazi | 4[20%] |
| Mixed | 4[20%] |
| Current smokers, n [%] | 2 [10%] |
| BMI (Kg/m2), median [IQR] | 24.7[20.7-26.1] |
| **Concomitant of diseases, n [%]** |  |
| Thyroid disease | 2 [10%] |
| Asthma | 2 [10%] |
| Other | 3 [15%] |
| **Concomitant medications, n [%]** |  |
| PPI | 1 [5%] |
| Levothyroxine | 2 [10%] |
| Other | 2 [10%] |
| **Dietary supplements, n [%]** |  |
| Vitamin D | 2 [10%] |
| Folic acid | 2 [10%] |
| Probiotic | 1 [5%] |
| B12 | 1 [5%] |
| Other | 3 [15%] |
| **Family history, n [%]** |  |
| Inflammatory bowel disease | 4 [20%] |
| Psoriasis | 1 [5%] |
| Rheumatoid arthritis | 2 [10%] |
| Asthma | 3 [15%] |
| Thyroid disease | 4 [20%] |
| Diabetes mellitus, type2 | 1 [5%] |
| Cancer, any type | 3 [15%] |

**Table S2: Mean dietary intake parameters of the study**

| **Dietary intake** | **Baseline** | **After MED intervention** | **P value** | **Recommended daily intake** |
| --- | --- | --- | --- | --- |
| **Total kcal** | 1962 (530) | 1928 (780) | 0.82 |  |
| **Protein (g/d)** | 88 (33) | 93 (44) | 0.64 |  |
| **Carbohydrate (g/d)** | 198 (61) | 180 (77) | 0.32 |  |
| **Fat (g/d)** | 86 (29) | 87 (46) | 0.91 |  |
| **Saturated fat (g/d)** | 27 (9) | 21 (6) | 0.03 | < 10% of kcal/ 22 g/d |
| **Cholesterol (mg/d)** | 335 (177) | 231 (156) | 0.07 | < 300 mg/d |
| **Dietary fiber (g/d)** | 24 (13) | 30 (13) | 0.008 | > 28 g/d |
| **Sodium (mg/d)** | 4070 (2590) | 2670 (1314) | 0.06 | < 2300 mg/day |

* Data is presented as mean (SD)

**Table S3: metabolic and inflammatory markers pre and post intervention**

| p | Post intervention (Week 4) | Baseline  (Week 0) |  |
| --- | --- | --- | --- |
| 0.61 | 87±42 | 92±45 | Triglycerides ((mg/dl), mean ± SD |
| 0.11 | 163±31 | 173±31 | Cholesterol ((mg/dl), mean ± SD |
| 0.44 | 84±30 | 84±34 | LDL ((mg/dl), mean ± SD |
| 0.18 | 62±15 | 65±16 | HDL ((mg/dl), mean ± SD |
|  | | | |
| 0.27 | 0.14 (0.06-0.26) | 0.09 (0.06-0.15) | CRP ((mg/dl), |
| 0.02 | 11 (6-33) | 16.5 (8-47) | Fecal calprotectin (mg/kg) |
|  | | | |
| 0.35 | 24.2 ±8 | 23.8±9 | Folic acid (nmol/L), mean ± SD |
| 0.2 | 318±138 | 330±151 | Vitamin B12 (pmol/L), mean ± SD |

**Supplementary Methods:**

DNA extraction and sequencing and processing of raw data

In brief, DNA was extracted using Magcore Nucleic Acid Extractor (RBC©) and the MagCore DNA Tissue kit according to the manufacturer’s instructions, with the following adaptations: 10-50 mg of sample were transferred to a tube containing glass lysis beads (Bead Beating Tube Type C [Soil]; Geneaid); the exact amount taken was recorded to allow absolute DNA quantification. Buffer GT from the Magcore DNA Tissue kit was added to the tubes, which were then placed in a Bead Beater (BioSpec, USA) for two minutes. After removal of lysis glass beads and cell debris by centrifugation, the lysate was incubated with Proteinase K, and further processed according to manufacturer’s instructions. Libraries were prepared using a two-step PCR protocol, with the 1^st^ step utilizing primers tailed with CS1/CS2 common sequences and targeting either the V4 region of 16S rRNA gene (515F: 5’-GTGCCAGCMGCCGCGGTAA , 806R: 5`-GGACTACHVGGGTWTCTAAT, 25 cycles) for bacterial workflow or region 1-399 of the 18S rRNA gene for eukaryotic workflow, as previously presented^1^ (5’-AAAGCCATGCATGYCTAAGTATMA, 5’-CTCAGGCTCCYTCTCCGG, 30 cycles). Access Array primers for Illumina (Fluidigm) were used in a second, 10 cycle PCR to add barcode, adaptor, and index sequences to each sample. Libraries were cleaned using Kapa Pure beads (Kapa), quantified by Qubit, and equimolar amounts were pooled and sequenced using Illumina Miseq v2 Kit (500 cycles) to generate 2x250 paired-end reads.

All scripts used to process this data were uploaded to: <https://github.com/leahfa/MED-microbial-analysis>. A custom R script was used to identify and remove sequencing primers, and DADA2^2^ R package was then used to process raw sequences to normalized taxonomy tables, using a standard workflow as demonstrated in https://benjjneb.github.io/dada2/tutorial_1_8.html. DADA2 processing commences with quality filtration, setting for this project maxEE (maximal expected errors allowed) to 2, and truncating sequence length at 210. Error learning, which is an estimation of error rate per sequencing run, dereplication, inference of unique Accurate Sequence Variants (ASVs), paired-end merging and chimera removal were all conducted with dada2 default settings. Taxonomic assignment was performed against Silva database v138. As the eukaryotic microbiome may also contain sequences originating in host or food, sequences assigned to the phyla Archaeplastida and Excavata, the class Metazoa, the genera *Ochromonas* (algae) and *Agaricus* (edible mushrooms), or these without any phylum-level assignment at all, were removed from the 18S dataset. Median sample depth at the end of the pipeline was 10594 seqs/sample for 16S microbiome, and 5400 seq/sample for 18S eukaryome. To avoid bias related to sequence depth, data were rarified to an equal depth of 5000 seq/sample (for 16S) and 1480 seqs/sample (for 18S; one sample which failed to reach this threshold was discarded).

Estimation of bacterial absolute abundances

Absolute amounts of bacterial DNA per sample were estimated using QPCR as presented in^3^. DNA samples were diluted x10 in water to preserve DNA quantity and reduce the possibility of PCR inhibition. 1 ul diluted DNA was added to PCRBIO SyGreen© master mix, and 40 PCR cycles (15' 95C, 15' 53C, 10' 72C) performed on BIORADs CFX Connect© using the same 16S primers as above. Three replicate wells were loaded per sample, with two NTC (no template controls) and a reference standard (serial dilutions of ZymoBIOMICS© Microbial Community DNA Standard, spanning 0.017 to 10 ngDNA/µl) loaded on each plate. DNA concentration per sample was calculated by the formula: DNA.conc=exp(α*Cq+β), where α and β are the slope and intercept coefficients derived from the standard curve loaded on that same plate. The total amount of DNA per sample (DNA concentration*extraction volume) was then normalized by the amount of input stool (mg) used for extraction, yielding absolute bacterial loads of DNA(µg)/Stool(mg).

References:

1. Dollive S, Peterfreund GL, Sherrill-Mix S, et al. A tool kit for quantifying eukaryotic rRNA gene sequences from human microbiome samples. *Genome Biol*. 2012;13(7):R60. doi:gb-2012-13-7-r60 [pii]\r10.1186/gb-2012-13-7-r60

2. Callahan BJ, Mcmurdie PJ, Rosen MJ, Han AW, Johnson AJ, Holmes SP. DADA2 : High resolution sample inference from amplicon data. *bioRxiv*. Published online 2015:0-14. doi:10.1101/024034

3. Contijoch EJ, Britton GJ, Yang C, et al. Gut microbiota density influences host physiology and is shaped by host and microbial factors. *Elife*. 2019;8:1-26. doi:10.7554/eLife.40553
